# Electrical behaviour of dendritic spines as revealed by voltage imaging

**DOI:** 10.1038/ncomms9436

**Published:** 2015-10-05

**Authors:** Marko A. Popovic, Nicholas Carnevale, Balazs Rozsa, Dejan Zecevic

**Affiliations:** 1 Department of Cellular and Molecular Physiology, Yale University School of Medicine, New Haven, Connecticut 06510, USA; 2 Institute for Multidisciplinary Research, Belgrade University, Belgrade 11030, Serbia; 3 Department of Neurobiology, Yale University School of Medicine, New Haven, Connecticut 06510, USA; 4 Institute of Experimental Medicine of the Hungarian Academy of Sciences, Budapest H-1083, Hungary; 5 The Faculty of Information Technology, Pázmány Péter University, Budapest H-1083, Hungary; 6 Kavli Institute for Neuroscience, Yale University School of Medicine, New Haven, Connecticut 06510, USA

## Abstract

Thousands of dendritic spines on individual neurons process information and mediate plasticity by generating electrical input signals using a sophisticated assembly of transmitter receptors and voltage-sensitive ion channel molecules. Our understanding, however, of the electrical behaviour of spines is limited because it has not been possible to record input signals from these structures with adequate sensitivity and spatiotemporal resolution. Current interpretation of indirect data and speculations based on theoretical considerations are inconclusive. Here we use an electrochromic voltage-sensitive dye which acts as a transmembrane optical voltmeter with a linear scale to directly monitor electrical signals from individual spines on thin basal dendrites. The results show that synapses on these spines are not electrically isolated by the spine neck to a significant extent. Electrically, they behave as if they are located directly on dendrites.

## Abstract (web summary)

Dendritic spines located on individual neurons process information, but our understanding of the electrical behaviour of spines is still limited. Here, the authors use voltage-sensitive dye imaging techniques to monitor electrical signals from thin basal spines and show that synapses are not electrically isolated by the spine neck.

## Graphical abstract

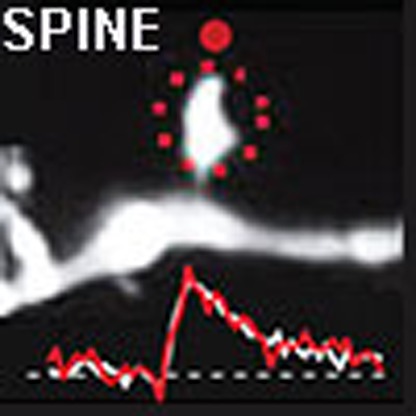

Spines are small (∼1 μm) membrane protrusions from dendrites which receive most of the excitatory synaptic inputs in the mammalian brain. They are of critical importance because they utilize a complex assembly of transmitter receptors and voltage-sensitive ion channel molecules^1^ to process electrical input signals and to mediate synaptic plasticity that may underlie learning and memory^2^. There is, however, no direct information on the electrical behaviour of spines because it has not been possible, for technical reasons, to measure the generation and spread of input signals from the spine to the dendrite. Theoretical considerations show that the main determining factor for the electrical role of dendritic spines is the electrical resistance of the spine neck (*R*_neck_) relative to the input impedance of the parent dendrite (*Z*_dendrite_)^3^,^4^,^5^,^6^,^7^. Obtaining direct evidence on these variables requires a method for monitoring subthreshold membrane potential responses simultaneously from the spine head and the parent dendrite as well as a technique for a selective activation of individual excitatory synapses on the spine head. Methods for selective activation of individual synapses have been developed using iontophoresis from sharp microelectrodes^8^ and later improved by two-photon uncaging of glutamate^9^,^10^,^11^. However, recording of subthreshold synaptic responses at the spatial scale of individual spines with the sensitivity and temporal resolution adequate for quantitative analysis has been a major challenge. A notable attempt to optically monitor subthreshold signals from individual spines^12^ was based on the realization that electrochromic voltage-sensitive dyes^13^ might be utilized to achieve that goal. However, due to low sensitivity of the recording method that study could not provide conclusive evidence.

Here, we used an advanced version of electrochromic voltage-sensitive dye technique^14^ which allowed us to directly measure subthreshold excitatory postsynaptic potential (EPSP) signals from individual spines and quantify electrical resistance of the spine neck. The EPSP signals were evoked by selective activation of individual synapses of layer 5 cortical pyramidal neurons of the mouse using focal glutamate release. The results demonstrated that synapses on spines of thin basal dendrites are not electrically isolated by the spine neck to a significant extent. Thus, in our measurements, spines with a variety of morphological features behaved the same in terms of electrical signalling. The optical approach described here lights the way to future studies of how particular combinations of transmitter receptors and voltage-sensitive ion channels in different spines^15^ act in concert to shape the integration of chemical input signals at the site of origin.

## Results

### Sensitivity of optical recording

**Figure 1:**
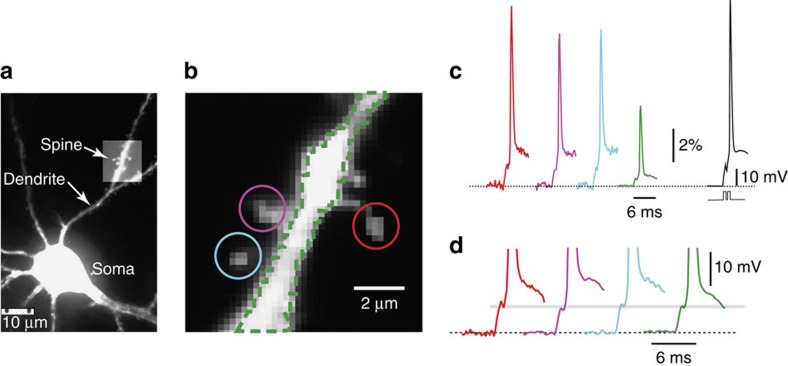
Sensitivity of recording. (**a**) Voltage-sensitive dye fluorescence image of a neuron; confocal, *z*-stack projection. Bright rectangle: illuminated recording region. (**b**) High magnification single frame image focused on the spine inside the red circle obtained with the CCD for voltage imaging. (**c**) Subthreshold and AP optical signals from colour-coded multiple locations (temporal average of 16 trials; spatial average of pixels within coloured outlines). Black traces: somatic patch electrode recording (upper trace); transmembrane current pulses (lower trace). (**d**) Optical signals calibrated in terms of membrane potential using bAP signal as calibration standard. The amplitude resolution was improved by additional temporal averaging (the thickness of the grey line is 1 mV; average of 80 trials). The differences in after-depolarization recorded from different locations are not reproducible and likely reflect interaction between slow noise and the exponential subtraction routine used to compensate for dye bleaching. The recording sensitivity shown in **c** was routinely achieved in all measurements (*n*=29).

A critical concern of this study was the sensitivity of optical recordings in terms of the signal-to-noise ratio (*S*/*N*) at the required spatiotemporal resolution. The best existing sensitivity which allowed optical monitoring of back-propagating action potentials (bAP) from dendritic spines^14^ was insufficient because EPSP signals would be 5–10 fold smaller in amplitude. The required improvement in sensitivity was accomplished by further increase in the excitation light intensity using a laser at the wavelength that has the best signal, by minimizing photodynamic damage by restricting the excitation light to a small area (18 μm × 18 μm), and by briefly lowering oxygen concentration in the extracellular solution during optical recording (Methods). The sensitivity of optical recording under these conditions is illustrated in Fig. 1. An image of a cortical layer 5 pyramidal neuron situated in the superficial layer of the slice (<30 μm from the surface) and labelled with the voltage-sensitive dye was projected onto a charge-coupled device camera (CCD) for voltage imaging at high magnification so that individual spines could be clearly resolved (Fig. 1b). We selected spines that were isolated from their neighbours both in *x*–*y* and in *z* dimension; the selection was biased against spines with small heads because these were characterized with poor signal-to-noise ratio. The range of distances from the soma along the basal dendrite was 30–120 μm. In a representative experiment shown in Fig. 1, a subthreshold depolarizing transient followed by an action potential were evoked by two current pulses delivered from a somatic patch electrode while optical signals were acquired at a frame rate of 2 kHz from a small segment of a basal dendrite with several spines. Both the subthreshold membrane potential signal and the action potential signal can be clearly resolved in optical recordings from the three spine heads and the parent dendrite with modest signal averaging (temporal average of 16 trials; Fig. 1c). As a rule, the amplitudes of optical signals related to the same electrical event are different when recorded from different neuronal compartments reflecting differences in recording sensitivity due to variability in the surface-to-volume ratio^16^. Thus, it is necessary to calibrate optical signals on an absolute scale (in mV) to compare signal amplitudes. This was accomplished by normalizing the subthreshold signals to an optical signal from a bAP^12^,^16^ which has a known declining amplitude along basal dendrites, as previously determined by patch-pipette recordings^12^,^17^. The subthreshold and AP optical signals can also be calibrated using long hyperpolarizing pulses delivered to the soma^12^,^18^, which attenuate relatively little as they propagate along dendrites^17^,^19^. Both methods of calibration have to be regarded as an approximation due to a known individual variability between neurons and possible inaccuracies in patch-pipette recordings from thin dendrites^20^. However, we determined that the margin of calibration error is sufficiently small that it does not influence the conclusions of our study (see below). Throughout this study, we used bAP signals as calibration standards because they are substantially shorter compared with steady state hyperpolarizing steps and, thus, less susceptible to slow noise, dye bleaching effects and photodynamic damage. We experimentally confirmed the result previously reported by Palmer and Stuart^12^ showing that both methods of calibration produce the same results (Supplementary Fig. 1, Methods). In the experiment shown in Fig. 1, the size of the AP measured with an electrode in the soma was 110 mV which corresponds to an average value of 80±10 mV bAP in the basal dendrite at a distance of ∼50 μm from the cell body^17^ where the spine was located; in all experiments, the bAP amplitude at a distance corresponding to spine position on the basal dendrite was used as a calibration standard. The subthreshold electrical signals that were initiated in the soma are expected to be identical in the three spines and the parent dendrite within the ∼10-μm long dendritic segment shown in Fig. 1. This is because both theory and experiments demonstrated that electrical signals do not attenuate as they propagate from the dendrite into the spines^4^,^12^,^14^. Calibrated subthreshold signals were indeed identical within a fraction of a millivolt as shown in Fig. 1d.

### Linearity and photodynamic damage

**Figure 2:**
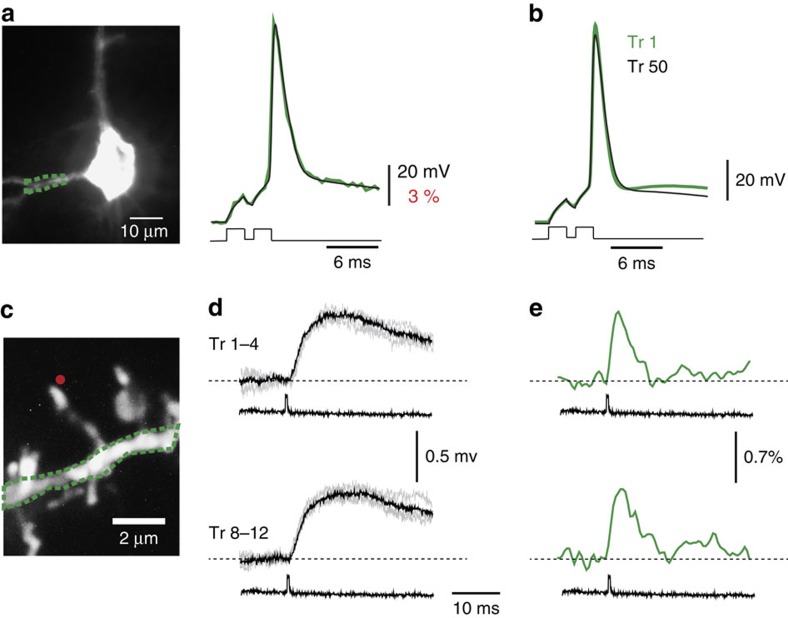
Linearity and photodynamic damage. (**a**) Superimposed electrical and optical recordings of an evoked bAP. Dye signal (green trace) from dendritic area outlined by green line is linearly related to membrane potential recorded with patch electrode (black trace). (**b**) The 1st and the 50th 20 ms optical recording trial of the evoked bAP signals from basal dendrite (spatial average of pixels within green outline). Photodynamic damage is small or absent. The small difference in AP size and shape is a common result of preparation rundown. (**c**) Single frame voltage-sensitive dye fluorescence image of a spine in recording position. Red dot: uncaging location. (**d**) Individual (grey) and average (black) responses of the first (1–4) and the last (8–12) eEPSP signals evoked by repetitive two-photon uncaging of glutamate as recorded by a somatic patch electrode. (**e**) Optical recordings of the same eEPSP signals from the outlined area (green line) on the parent dendrite at the base of the spine. Optical signals (green line) are average of four trials. Photodynamic damage is not detectable. Linearity and absence of photodynamic damage has been confirmed in all experiments (*n*=29).

Correct interpretation of optical recordings of membrane potential transients depends on two basic requirements: (a) light intensity has to be linearly proportional to membrane potential over the range of signal amplitudes; (b) to allow signal averaging, the electrical response under study has to be stable throughout the experiment indicating the absence of photodynamic damage. We confirmed that both requirements were met (Fig. 2, Methods).

### Spatial resolution

**Figure 3:**
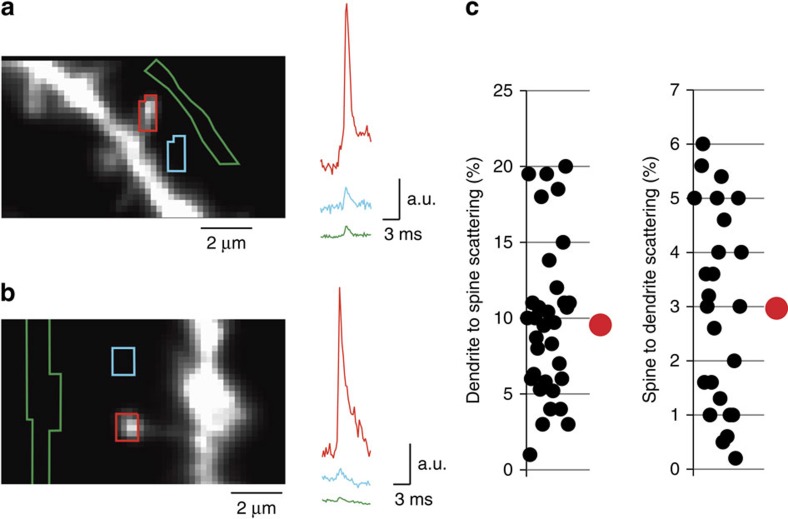
Light scattering. (**a**) Optical signals related to an evoked bAP from a stubby spine head and from an unstained area receiving scattered light. Signals are from areas outlined by corresponding colour lines. The ratio of the spine signal amplitude (red trace) and the amplitude of the signal from an area without spine (blue trace) is a measure of the amount of light scattering from the dendrite to the spine. The ratio of the spine signal amplitude (red trace) and the amplitude of the signal from an unstained area (green trace) at the distance from the spine head equal to the distance of the parent dendrite is a measure of the amount of light scattering from the spine to the dendrite. (**b**) Same test for a long neck mushroom spine. (**c**) Scatter plots and mean values for scattered light contribution to dendritic and spine head signals; summary data from *n*=29 experiments. Standard errors of the mean are smaller than red symbols.

Another critical parameter is the spatial resolution that can be achieved in wide-field epifluorescence microscopy mode, which has to be adequate to allow recording of spine and dendrite signals separately. To maximize spatial resolution, all measurements were made from spiny basal dendrites in the superficial layer of the slice (Methods). However, because signal contamination due to light scattering depends not only on recording depth but also on other structural and geometrical factors, the extent of light scattering was determined in every experiment. The amount of light scattered from the spine head to the dendrite was estimated by comparing bAP signals from the spine head and from an unstained area equivalent in size to the parent dendrite and at the same distance from the spine (Fig. 3a,b). The amount of light scattered from the dendrite to the spine head was estimated by comparing recordings from the spine head and from an analogous location at the same distance from the dendrite which contained no stained structures (Fig. 3a,b). The summary result from 29 spines analysed in this study (Fig. 3c) indicates that the amount of light scattered from the spine to the dendrite was negligible (3±0.3%; median 3%, range 0.2–6%) as expected from the ratio of membrane surface areas. The amount of light scattered from the dendrite to the spine was 10±0.9% (median 9.7%; range 1–20%). It is possible to estimate whether this amount of scattering will cause significant change in the EPSP signal recorded from the spine head. If one makes an assumption that the amplitude of the true EPSP related fractional signal (Δ*F*/*F*) from the spine head is equal to 1 and the one from the dendrite is equal to 0.5 (a hypothetical attenuation across the spine neck of 50%), the recorded optical signal will be composed of 90% of the light carrying the fractional signal from the spine head equal to 1 and 10% of the light carrying the fractional signal from the dendrite equal to 0.5. The recorded composite fractional signal will be 0.95, a 5% error from the true EPSP amplitude of 1 in this example. Even for the extreme (unrealistic) attenuation of EPSP amplitude across the spine neck of 99%, the upper bound for the error would be <10%. It will be shown below that, in our measurements, the actual error due to light scattering has to be much less than 5% and, thus, negligible. This is because the recorded amplitudes of the EPSP signals, even assuming an error of 10%, indicated that the attenuation across the spine neck is ∼10%, considerably less than a hypothetical value of 50% used above.

### Selective activation of individual spines

The analysis of EPSP signals from spines requires selective activation of individual synapses to eliminate uncertainties about the source of the signal. A reliable selective activation of one spine/synapse using physiological synaptic stimulation is not presently realizable. While it is possible, although difficult, to insure a reliable repetitive activation of only one individual presynaptic axons, one cannot assume that no more than one synapse is activated^12^. It is known that practically every presynaptic axon makes more than one functional contact (synapse) with any postsynaptic neuron. Thus, even a minimal stimulation of one presynaptic axon will result, as a rule, in activation of multiple synapses^12^,^21^.

**Figure 4:**
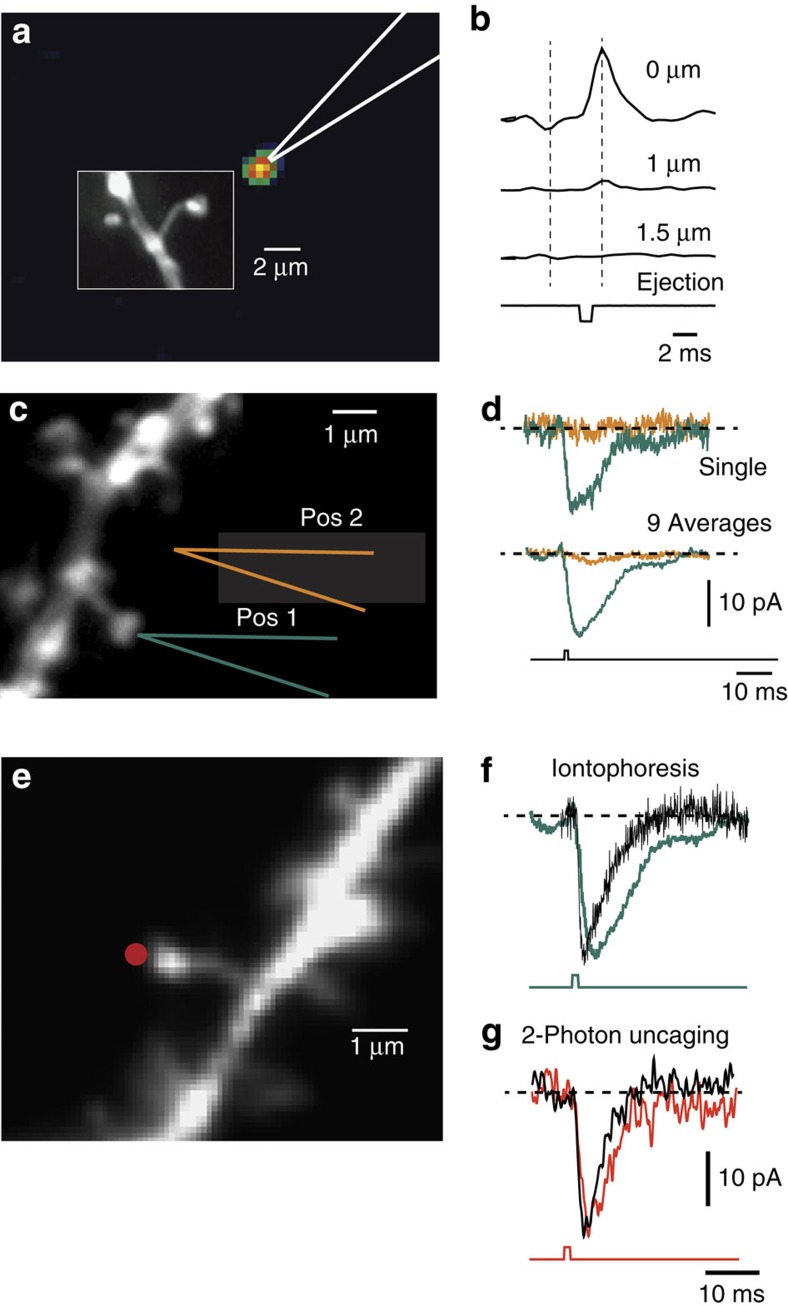
Spatiotemporal resolution of glutamate release. (**a**) A typical release profile with sub micrometre resolution of iontophoresis of a fluorescent dye with molecular weight similar to glutamate. (**b**) Light intensity as a function of distance from the tip of the sharp electrode. (**c**,**d**) An excitatory postsynaptic current of 15 pA recorded from the soma following focal glutamate iontophoresis onto an individual spine head. Repeated iontophoresis produced consistent responses; compare single trial and average of nine trials. Glutamate selectively activated the synapse on the spine head; placing the electrode at the same distance from the dendrite but away from the spine head resulted in no measurable response. This control measurements were carried out in *n*=19 experiments. (**e**) Fluorescence image of a spine positioned in close proximity (∼0.5 μm) to the uncaging site. (**f**) Synaptic current response to iontophoretic release of glutamate (green trace) adjusted to approximate the amplitude and time course of a spontaneous miniature EPSC (black trace). (**g**) Synaptic current response to two-photon uncaging of glutamate (red trace) adjusted to approximate the amplitude and time course of a spontaneous miniature EPSCs (black trace). The average 10–90% rise time and full width at half height (FWHH) of the spontaneous EPSC recorded in the soma were 1.2±0.1 ms and 5.9±0.4 ms, respectively (*n*=8). Corresponding values for uncaging-evoked responses were 2.1±0.2 ms and 6.1±0.3 (*n*=10) while EPSC evoked by iontophoresis where characterized with rise time of 2.9±0.2 ms and FWHH of 12±0.6 ms (*n*=19).

To insure that no more than one spine is activated we initially took advantage of micro-iontophoresis of glutamate onto individual spines from a high-resistance sharp electrode as developed in cell culture^8^. We extended this method to brain slices and confirmed the previously demonstrated spatiotemporal resolution (Fig. 4, Methods). However, micro-iontophoresis suffers from inherently low success rate (∼5%) in experiments that required multiple and reproducible activation of synapses because high-resistance electrodes are notoriously unstable. Thus, in later experiments, we used photolysis of caged glutamate in a diffraction-limited volume based on two-photon light absorption. The firmly established^9^,^10^,^22^ diffraction-limited spatial resolution of two-photon glutamate uncaging was verified in our measurements (Supplementary Fig. 2, Methods). Glutamate release using both iontophoresis and two-photon uncaging was standardized to evoke EPSPs in the soma in the range of 0.2–0.8 mV. These values correspond well to somatic recordings of physiological unitary EPSPs under optically confirmed activation of one individual spine on a neuron^21^.

### Electrical coupling across the spine neck

**Figure 5:**
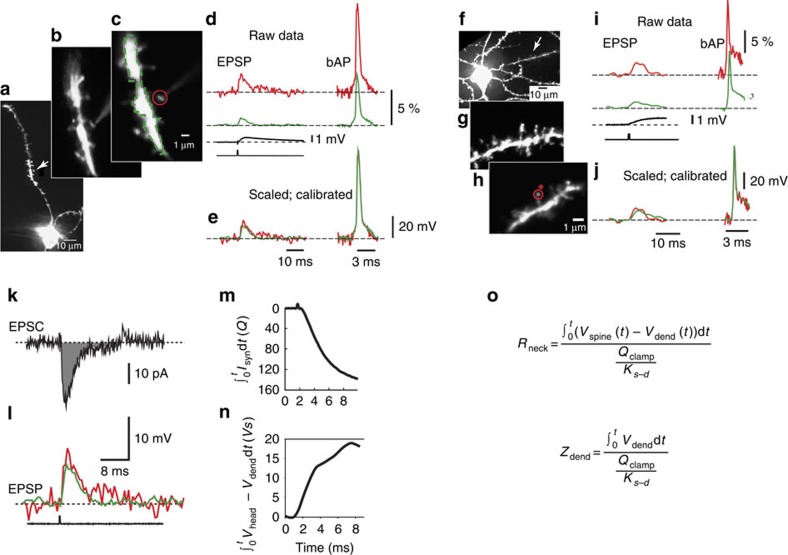
Quantification of *R* _neck_ and *Z* _dendrite_ from two representative experimental measurements. (**a**) Low-magnification fluorescence image of a basal dendrite labelled with a voltage-sensitive dye; *z*-stack of confocal images. Arrow: recorded spine. (**b**) High magnification confocal image of the same spine. Tip of iontophoretic electrode (labelled with the fluorescent dye) in the immediate vicinity of spine head. (**c**) Single frame image of a spine in recording position obtained with CCD for voltage imaging. (**d**) Traces on left: eEPSP recordings from spine head (red) and parent dendrite (green). Average of 16 trials. Bottom black traces: somatic electrode recording and the uncaging command pulse. Traces on right: bAP signals from same locations. Average of nine trials. (**e**) Left traces: superimposed eEPSP signals from spine head and parent dendrite calibrated in terms of membrane potential. Right traces: bAP signals corrected for recording sensitivity difference. (**f**–**j**) Two-photon uncaging of glutamate. Same information as shown in **a**–**e**. Red dot in **h**: position and approximate size of uncaging light spot. The eEPSP and bAP recordings are average of 8 and 4 trials, respectively. (**k**) Synaptic current in response to standard focal application of glutamate. Grey area: time integral of synaptic current. (**l**) Superimposed eEPSP signals from spine head (red) and parent dendrite (green) calibrated in mV. (**m**) Time integral of synaptic current. (**n**) Time integral of voltage drop across spine neck. (**o**) Equations for *R*_neck_ and *Z*_dendrite_ calculation. *V*_dendrite_—local dendritic membrane potential; *V*_spine_—membrane potential of the spine head; *I*_synapse_—synaptic current; *R*_neck_—electrical resistance of spine neck; *Z*_dendrite_—impedance of parent dendrite; *Q*_clamp_—total recorded charge transfer; *K*_s-d_: distance-dependent adjustment factor for the correction of the somatic voltage clamp error based on experimentally determined dendro-somatic attenuation of synaptic charge transfer^23^.

**Figure 6:**
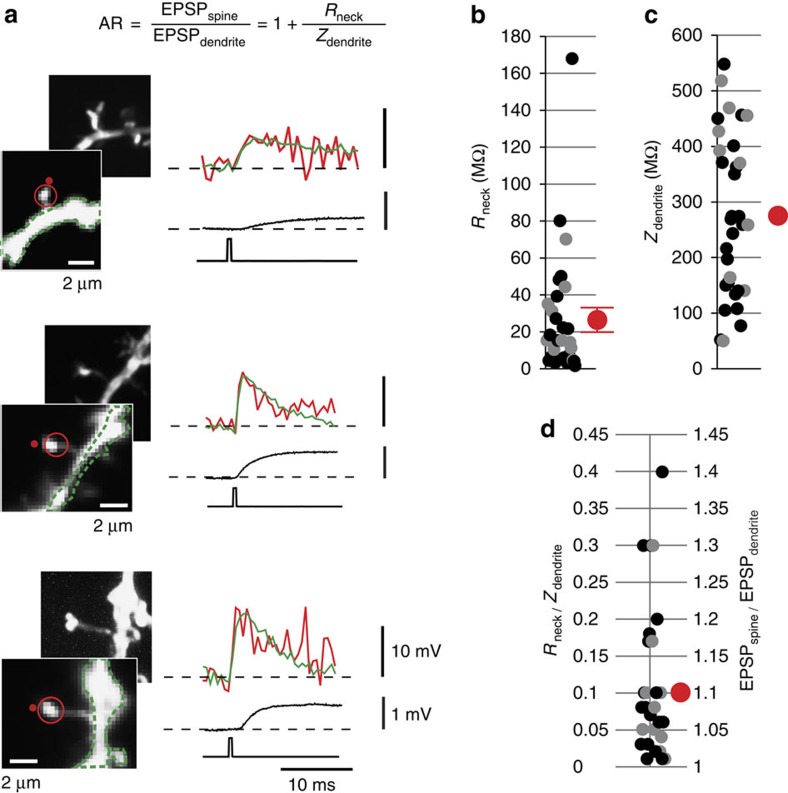
Attenuation ratio eEPSP_spine_/eEPSP_dendrite_. (**a**) The attenuation ratio is directly determined by the ratio of resistances as shown by the equation at the top. Three representative examples of the comparison of eEPSP_spine_ (red) and eEPSP_dendrite_ (green) evoked by two-photon uncaging of glutamate. In each panel, two fluorescent images are shown on left. Upper: *z*-stack of confocal images. Lower: single frame image of a spine in recording position. Red dot: uncaging location. Lower black traces: somatic patch electrode recordings and timing of uncaging pulse. From top to bottom: averages of 24, 16 and 4 trials. (**b**) Scatter plot of individual values and the mean *R*_neck_. (**c**) Scatter plot of individual values and the mean *Z*_dendrite_. (**d**) Scatter plot of individual values and the mean ratios *R*_neck_/*Z*_dendrite_ (left scale) and eEPSP_spine_/eEPSP_dendrite_ (right scale). *N*=29 in all cases. In **c** and **d** s.e.m. is smaller than the red data mark. (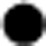) Iontophoresis. (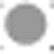) 2P uncaging.

To characterize electrical coupling across the spine neck we monitored evoked subthreshold signals (eEPSP) following brief focal application of glutamate. A representative experiment utilizing micro-iontophoresis is shown in Fig. 5a–e. The tip of the sharp electrode was positioned in close proximity to a spatially isolated spine (Fig. 5a–c) and the iontophoretic current was adjusted so that the eEPSP in the soma (Fig. 5d) was in the range of unitary synaptic events (0.2–0.8 mV). The eEPSP signal was recorded optically from the spine head and from the parent dendrite followed by the recording of the bAP signals from the same locations (Fig. 5d). The EPSP signals were calibrated in mV using the bAP signal as a calibration standard (scaled signals in Fig. 5e). The significance of possible errors in calibrating optical signals is discussed below. From the measured eEPSP_spine_, eEPSP_dendrite_ and *I*_synapse_ (recorded separately under voltage clamp in response to a standard glutamate release adjusted to evoke EPSPs in the soma corresponding to somatic recordings of physiological unitary EPSPs) we calculated *R*_neck_ and *Z*_dendrite_ (Fig. 5k–m) according to the equations in Fig. 5o applied to the equivalent electrical circuit shown in Supplementary Fig. 3b. The equations in Fig. 5o are derived as time integrals of Ohm’s law applied to a voltage divider representing a spine attached to a dendrite (Supplementary Fig. 3c). This approach integrated synaptic current (*I*_synapse_) over time to arrive at the recorded synaptic charge transfer (*Q*_clamp_) which was subsequently corrected for the error caused by incomplete space clamp. Integrating synaptic current to estimate transferred charge minimizes somatic voltage clamp error^23^. The significance of this error is discussed below. In the experiment shown in Fig. 5a–e, *R*_neck_ and *Z*_dendrite_, calculated from measured parameters, were 15 and 197 MΩ, respectively. Figure 5f–j shows an example of a similar experiment utilizing two-photon uncaging of glutamate. Again, it is clear that the eEPSP signals in the spine head and in the parent dendrite (Fig. 5i) are similar indicating that *R*_neck_ is negligible compared with *Z*_dendrite_. In this experiment *R*_neck_ and *Z*_dendrite_, calculated from the measured parameters, were 29 and 371 MΩ, respectively. Figure 6 illustrates three additional measurements utilizing two-photon uncaging. The scatter plots of data from *n*=29/24/22 (spines/cells/animals) experiments showed the mean values of *R*_neck_=27±6 MΩ and *Z*_dendrite_=275±27 MΩ.

### Quantification accuracy

How accurate is our quantification of *R*_neck_ and *Z*_dendrite_? There are four possible sources of errors that need to be considered: (a) sampling frequency, (b) the *S*/*N* ratio, (c) calibration of optical signals in terms of membrane potential and (d) voltage-clamp measurement of unitary synaptic current/transferred charge. The sampling frequency (frame rate) was limited by the available *S*/*N* to 2 kHz. The duration of the upstroke of the EPSP (threshold-to-peak) as recorded electrically by others at a sampling rate of 50 KHz is in the range of 1–2 ms (refs 17, 23, 24, 25). In our study, the average 10–90% rise time of optically recorded eEPSPs was 1.2±0.1 ms while the full width at the half height was 5.9±0.4 ms; *n*=29. Our sampling rate of 2 kHz, although lower than the formally required Nyquist rate, was sufficient for the accurate reconstruction of EPSP waveform because aliasing (sampling artefact which can result in the omission of additional peaks in between data points) can be safely excluded on the basis of additional information available from independent measurements; the general shape of the EPSP is known from dendritic electrical recordings^25^. These electrical data correspond well with our optical recordings from the parent dendrite.

At the sampling rate of 2 kHz, optical recordings were averaged over 4–16 trials (and rarely up to 25 trials) to reach the *S*/*N* of ∼4. The mean *S*/*N* in recordings from *n*=29 spines was 4.3±0.3 (mean±s.e.m.). Due to the larger membrane area, the sensitivity of recordings from the parent dendrites at the base of the spine was significantly higher with the *S*/*N*=9.4±2.3. We assumed that the high sensitivity recordings from the parent dendrite are an accurate measure of the true waveform of the eEPSP both in the parent dendrite and in the spine head because the waveform is expected to be practically identical in both compartments. The membrane area of the spine neck and its effective capacitance is extremely small rendering RC filtering across the ∼1 μm long spine neck cable negligible. In addition, regarding the *S*/*N*, the calculation of the *R*_neck_ and *Z*_dendrite_ is based on the time integral of EPSP signals from spines and dendrites. As illustrated in Fig. 5m,n, integration of the EPSP voltage trace over time acts as a powerful low-pass filter which practically eliminated random high-frequency noise. Thus, we conclude that the quantification of *R*_neck_ and *Z*_dendrite_ was based on adequate sensitivity and sampling rate.

There are unavoidable inaccuracies in calibrating optical signals in terms of membrane potential. However, it is possible to determine calibration error limits and establish whether the results and conclusions of the study could be significantly affected. The amplitude of the bAP signals measured electrically from basal dendrites^12^,^17^ varied from ∼100 mV at a distance of about 30 μm down to ∼45 mV at a distance of about 120 μm from the soma. Within this range, at any given distance, individual values may vary around the exponential fit to the data by ∼20 mV (ref. 17). Additionally, there are uncertainties about the precision of electrical measurements from thin dendrites which are often ignored. The accuracy of electrical measurements using high-resistance patch electrodes (∼100 MΩ) depends steeply on series resistance and capacitance compensation (which are never perfect). Moreover, patch electrodes introduce additional capacitive load on the dendrite resulting from the capacitance of the pipette that cannot be compensated for (ref. 20). These factors would lead to underestimation of the extent of AP backpropagation. Indeed, voltage-imaging studies showed that the amplitude of bAPs in distal basal dendrite might be larger than what was indicated by electrode measurements^26^. To take into account these uncertainties, we calibrated EPSP signals for all spines (regardless of the distance from the soma) using both the low-end of the bAP amplitude range (45 mV) and the high-end value of 100 mV. The results showed that the mean *R*_neck_ values based on low- and high-end bAP amplitudes were 22.5 ±4.8 MΩ and 43.7±9.7 MΩ, respectively. This result sets the upper and lower limits for *R*_neck_ values indicating that possible errors in calibrating optical signals are too small to influence the conclusions from this study. The mean value of 26.7±6.3 MΩ, obtained by adjusting bAP amplitude as a function of distance from the soma according to the exponential fit to individual data^12^,^17^ appears to be the most accurate estimate that can be currently obtained.

Due to incomplete space clamp, the somatic voltage clamp does not report accurate synaptic currents or transferred charge. To minimize these errors, we used the correction factors taken directly from Fig. 3a of Williams and Mitchell^23^. That figure reports the experimentally determined dendro-somatic attenuation of transferred synaptic charge as a function of distance of the synapse from the recording site (cell body) expressed as a fraction of recovered charge. In every experiment, we used the value of this fraction at the corresponding distance (as determined from the confocal image of the cell) as the correction factor (*K*_s-d_). The range of distances in our experiments was 30–120 μm (Methods) corresponding to a fraction of recovered charge in the range of 75–95%. However, the direct measurements of dendro-somatic attenuation by Williams and Mitchell^23^ were carried out from primary apical dendrites. Thus, our calculations of the synaptic currents are likely to be underestimates because apical dendritic trunks are characterized by larger diameter and, hence, lower axial resistance compared with basal dendrites. For this reason the charge loss is expected to be larger in basal dendrites. We conclude that our results set the lower limit for current amplitudes at the site of origin which translates into upper bound for calculated spine neck resistance.

It is important to recognize that, in addition to being small, the errors in calibrating optical signals as well as in estimating current amplitudes have no effect on the ratio of resistances because both resistances are equally affected. This is of particular significance because, from the functional point of view, the absolute values of the *R*_neck_ and *Z*_dendrite_ are less important than the ratio of resistances which directly determines the attenuation ratio eEPSP_spine_/eEPSP_dendrite_ (AR) (Fig. 6a; Supplementary Fig. 3). The AR, in turn, determines the effect of a synapse on the membrane potential of a neuron (synaptic weight). It follows from these considerations that AR can be measured directly from optical recordings without any assumptions and with accuracy limited only by the *S*/*N*. The scatter plot of the data from 29 experiments indicated mean value for AR=1.10±0.02 (Fig. 6d). In other words, on average, eEPSPs in the spine head are attenuated as they propagate across the spine neck by ∼10% with a significant proportion of spines (∼60%) showing lower attenuation.

### The role of voltage-sensitive channels in spines

**Figure 7:**
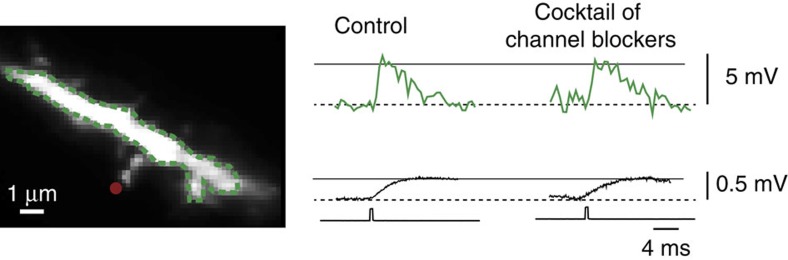
A representative example of the effect of channel blockers on eEPSP amplitude. Left panel: fluorescence image of a section of basal dendrite with one spine in focus; uncaging location indicated by red dot. Right panel: top traces: optical recordings of the eEPSP_dendrite_ signals from outlined dendritic area (green) evoked under control conditions and in the presence of a cocktail of channel blockers (1 μM TTX, 100 μM AP-5, 10 μM nimodipine, 100 μM NiCl_2_). Average of 4 trials. No effect of channel blockers detected. Middle traces: somatic electrode recordings of eEPSP. Bottom traces: timing of the uncaging pulse.

The conceptual model used for *R*_neck_ calculation (Supplementary Fig. 3) is valid only if voltage-sensitive channels in the parent dendrite do not contribute significantly to eEPSP_dendrite_ amplitude. We used a pharmacological test to measure this contribution (which is currently unsettled^12^,^27^,^28^). The eEPSP amplitudes were measured under control conditions and in the presence of a cocktail of pharmacological agents that selectively block NMDA receptors as well as Na^2+^ and Ca^2+^ voltage-sensitive channels (Fig. 7, Methods). In these measurements, it was sufficient to monitor eEPSP signals from a small section (∼20 μm in length; Fig. 7) of the dendrite at the base of the spine. These signals provide accurate information about changes in the synaptic current and eEPSP amplitude while the recording from the relatively large dendritic membrane area relaxes the requirement for signal averaging. The summary result from *n*=7/6/6 experiments (spines/cells/animals) showed that the pharmacological block of voltage-sensitive channels did not reduce the amplitude of the eEPSP (eEPSP_control_/eEPSP_blockers_=1.006±0.05; *P*<0.01; Student’s *t*-test). These data argue that the contribution of voltage-sensitive channels to unitary eEPSPs was not significant.

### Computational modelling

**Figure 8:**
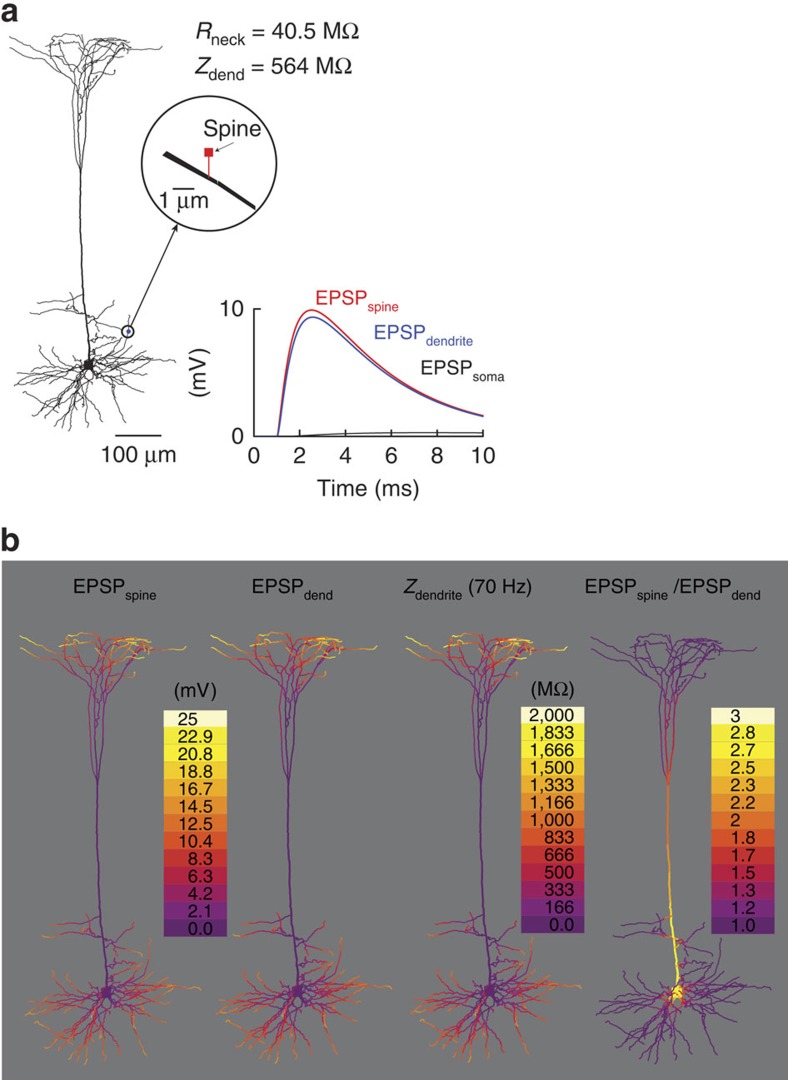
Numerical simulation. (**a**) Activated synapse on a spine attached to a basal dendrite 120 μm from the soma generated EPSP signal of 10 mV in the spine head, 9.3 mV in the parent dendrite, and 0.3 mV in the soma. The best fit of the size and shape of the EPSP signal corresponded to *R*_neck_=40.5 MΩ and *Z*_dendrite_=564 MΩ. (**b**) A series of simulations with EPSP_spine_ and EPSP_dendrite_ computed for a standard spine attached to all dendritic compartments (one at a time). Colour-coded display: spatial distributions of Z_dendrite_, EPSP_spine_, and EPSP_dendrite_ are similar while AR was close to unity for the majority of synapses in the dendritic tree.

To establish how our experimental results correspond to widely accepted electrical behaviour of dendritic cables we constructed a multicompartmental model of a layer 5 pyramidal neuron with typical passive dendritic cable properties (Methods) and experimentally determined dendritic diameters from live neurons (Supplementary Fig. 4). The model predicts the amplitude and the time course of EPSP_spine_, EPSP_dendrite_ and AR=1+*R*_neck_/*Z*_dendrite_ (refs 3, 4, 5, 6) for a given *I*_synapse_, according to Ohm’s law and Kirchhoff’s current law for spines at different locations (Fig. 8; Supplementary Fig. 3). In a series of simulations, the position of a typical spine (neck length 1 μm; diameter 0.17 μm)^7^ attached to a parent dendrite was moved across the entire dendritic arbour. The EPSP_spine_, EPSP_dendrite_, *Z*_dendrite_ and AR were calculated for every location after the spine was activated with a conductance change synapse model adjusted to mimic a unitary EPSC^21^. The colour-coded display of the results illustrates spatial distribution of the four parameters mentioned above (Fig. 8b). Because the calculated *Z*_dendrite_ was much larger than *R*_neck_ in most parts of the dendritic tree (a fact that is not universally appreciated^29^), the AR was close to unity (between 1 and 1.1) in these distal regions (Fig. 8b, right-most model result). For a typical basal dendrite the model predicted a ratio of 1.5, 1.3 and <1.1 at distances of 20, 30 and >60 μm from the soma. This modelling prediction corresponds well to the range of recorded values shown in Fig. 6d. The exception from this rule was the proximal primary dendritic trunk characterized by large diameter and a very low *Z*_dendrite_, comparable to the value of *R*_neck_ of the standard spine (∼30 MΩ). The AR in these proximal regions varied between 1.5 and 3 indicating that the EPSP in these spines was up to 3 times larger than in the dendrites at the base of the spine. However, larger attenuation in these spines is based on low *Z*_dendrite_, not on large *R*_neck_. Thus, the overall input impedance of a proximal spine (*Z*_spine_=*R*_neck_+*Z*_dendrite_) is low and, consequently, the peak amplitude of EPSPs produced in these spines is expected to be low. Patch-clamp recordings from basal dendrites confirmed this expectation (Nevian *et al*.^17^). Notably, these proximal dendritic compartments are largely devoid of spines^30^. Another consequence of much larger *Z*_dendrite_ relative to *R*_neck_ over most of the dendritic tree was that the spatial distribution of EPSP_spine_ amplitudes was highly non-uniform and similar to the spatial distribution of both *Z*_dendrite_ and EPSP_dendrite_ amplitudes (Fig. 8b, left three model results). This result shows that, in the model, *Z*_dendrite_ is the main determinant of the EPSP amplitude in both compartments (spine and dendrite) while the effect of *R*_neck_ is small and limited to proximal parts of the primary apical dendrite. Our experimental measurements are in full agreement with these modelling predictions.

## Discussion

On the conceptual level, a key question that has not been answered is whether the hypothetical electrical isolation of synapses on spine heads caused by a narrow spine neck is responsible for specific functions which are not supported by synapses on dendrites. Current interpretations of indirect morphological evidence^7^,^31^,^32^, optical recordings of diffusional exchange across the spine neck^7^,^32^,^33^,^34^,^35^, optical recordings of membrane potential and [Ca^2+^]_*i*_ signals^6^,^12^,^32^,^36^, and modelling studies^4^,^5^,^6^,^28^ have not provided a consistent and unifying description of the electrical behaviour of spines.

We utilized a technique based on an electrochromic voltage-sensitive dye which can be thought of as a transmembrane optical voltmeter with a linear scale capable of monitoring simultaneously electrical signals from individual spines and parent dendrites. Our study provides direct evidence that the resistance of the neck in spines on thin basal dendrites is too low to electrically isolate synapses on spine heads to an extent that would have functional consequences. In other words, in strictly electrical terms, the experimental data argue that synapses on spines of thin basal dendrites behave as if they are located directly on dendrites. We also found that the contribution of voltage-sensitive channels to unitary eEPSPs was not significant in these spines. Interestingly, a recent study demonstrated that this conclusion cannot be extrapolated to a specific class of spines found on granule cells of the olfactory bulb^28^. A fundamental implication of our findings is that spines on thin basal dendrites are characterized by uniform electrical behaviour regardless of considerable short-term and long-term variations in their morphology^7^,^31^,^29^.

The average value of *R*_neck_ determined in our experiments (∼30 MΩ) is in full agreement with pioneering^35^ as well as several subsequent estimates from measurements of diffusional equilibration across the spine neck in the majority (∼95%) of spines under physiological conditions^7^,^32^,^33^,^34^. A recent estimate of the spine neck resistance, based on supra-resolution (STED) morphological data and diffusional equilibration measurements using fluorescence recovery after photobleaching (FRAP) technique, indicated *R*_neck_ value centred around 56 MΩ (ref. 7). Allowing for the uncertainties about the true values for both the resistivity of the intracellular medium and the diffusion coefficients, that estimate of *R*_neck_ is in good agreement with our measurements. Additional strong evidence in support of low *R*_neck_ is a striking lack of correlation between spine neck lengths (varying within a wide range of 0–10 μm) and the EPSP rise time and associated Ca^2+^ signal in the spine head, as recently reported for exceptionally long spines on granule cells of the olfactory bulb^28^. Similar study based on morphological STED data and diffusional FRAP measurements from spines of CA1 pyramidal neurons reported the same lack of correlation between spine dimensions (neck length and diameter) on one hand and evoked EPSPs in the soma and Ca^2+^ transient in the spine head on the other hand^32^. These results are in agreement with the main implication of our study that electrical behaviour of spines is independent of their morphology. The reported lack of correlation is expected if *R*_neck_ varies with neck dimensions and diffusional resistance (as governed by elementary principles of chemical diffusion and electrical current flow) but stays within a range of values that are much smaller relative to *Z*_dendrite_ (except in proximal thick dendrites characterized by low *Z*_dendrite_). The agreement between our voltage-imaging results and prior diffusional measurements is critical because, due to the close analogy between diffusional and electrotonic coupling across the spine neck, it is difficult to argue against strong implications of diffusional measurements for the upper bound of the possible electrical resistance of the spine neck. In this context, it should be emphasized that there is strong evidence for a small percentage of spines (∼5%) that show high diffusional isolation of the spine head^7^,^33^,^34^. These exceptional spines are currently not well understood.

The only, to our knowledge, prior voltage-sensitive dye study of subthreshold signals from dendritic spines^12^ reported that it was difficult to detect spine voltage responses in the majority of spines while a relatively small subset of spines (∼18%) were characterized by responses that ranged from 0 to 25 mV. In a larger subset of spines (82%) no clear responses could be detected, a result consistent with low spine neck resistance. The comparison of this study with our data is difficult due to several technical limitations. First, the study of Palmer and Stuart^12^ did not selectively activate individual synapses on spines. Therefore, it was not possible to arrive at accurate information on the spine neck resistance or the attenuation of the EPSP across the spine neck. As stated in their paper, their extracellular stimulation activated multiple synaptic inputs resulting in an average somatic EPSP of ∼5 mV. A somatic EPSP of this magnitude corresponds to activation of 6–25 synapses^6^,^24^ generating background depolarization from inputs other that the activated spine. This background depolarization of unknown amplitude will spread instantaneously from the parent dendrite into the spine head without any voltage loss making it impossible to know isolated spine response. In this situation the result of the described subtraction of the response in the parent dendrite from the spine response is undefined. Additionally, Palmer and Stuart^12^ selected a small subset of spine recordings with the largest observed responses to estimate, in a computer model, that the upper bound for the spine neck resistance was ∼500 MΩ. They went on to show, in a model, that even an extreme resistance of 500 MΩ is not expected to cause significant synaptic saturation which would modify the somatic EPSP. Thus, similar to our results, their estimates ruled out significant electrical isolation of synapses on dendritic spines.

There is a discrepancy between our voltage-imaging measurements and the interpretations of indirect evidence from several experimental studies based on optical recording of intracellular calcium concentration changes^6^,^29^,^36^,^37^ which had suggested high electrical isolation of synapses by the spine neck. High electrical isolation of synapses on spines, in turn, had been hypothesized to reduce location-dependent variability of local EPSP amplitude and kinetics and to standardize synaptic activation of NMDA receptors and voltage-gated channels thereby promoting nonlinear dendritic processing and associated forms of plasticity and storage^5^,^6^,^29^,^38^. The recent study of Harnett *et al*.^6^ based on calcium imaging indicated *R*_neck_ of ∼500 MΩ, an order of magnitude higher than our estimate. It should be emphasized that the pattern of the colour-coded spatial distribution of *Z*_dendrite_ and AR reported in that study (Fig. 3 in Harnett *et al*.^6^) is similar to our modelling result. This similarity is expected because any model based on a constant spine neck resistance and variable dendritic input impedance will result in a spatial pattern of AR which follows the classical spatial distribution of local dendritic input impedance. This outcome is independent of the absolute value of the *R*_neck_. At the moment, there is no clear explanation for the discrepancy in estimating *R*_neck_. Calcium concentration changes are indirect, slow, and highly nonlinear indicator of transmembrane voltage changes. Additionally, the nonlinear relationship between calcium signals and transmembrane voltage is unstable due to high sensitivity of the state of calcium channels to the history of the resting membrane potential. Thus, it is important to verify the conclusions of studies based on calcium imaging by a more direct approach especially because these conclusions dramatically contradict diffusional resistance measurements as well as the predictions from classical biophysical modelling of dendritic cables. As stated above, an additional strong argument against high spine neck resistance are the two most recent studies based on Ca^2+^ measurements that failed to find significant correlation between the dimensions of the spine neck and uncaging-evoked EPSPs and Ca^2+^ transients^28^,^32^.

To summarize, direct measurements of *R*_neck_ and *Z*_dendrite_ reported here argue against high *R*_neck_ for the spines on thin basal dendrites and against all of the hypothetical functional implications of the electrical isolation of synapses on these spines. It remains to be investigated whether spines on other parts of the dendritic arbour and in other neuronal types behave in the same manner. The optical approach described here provides a path for these future studies. More generally, we predict that the voltage-imaging approach will be instrumental in facilitating the answer to the central question in spine physiology: what is the mechanism by which complex ensembles of specific transmitter receptors and voltage-sensitive ion channels localized to spine heads act in concert to shape the plastic integration of repetitive chemical input signals carried out by dendritic spines.

## Methods

### Slices, patch-clamp recording and intracellular application of dyes

All surgical and experimental procedures were performed in accordance with Public Health Service Policy on Humane Care and Use of Laboratory Animals and approved by Yale University Institutional Animal Care and Use Committee. Experiments were carried out on somatosensory cortex slices from 18 to 30-day-old mice of either sex. In about 50% of the experiments we used wild-type mice (Swiss Webster (CFW), Harlan Laboratories, Indianapolis); the remainder of the data were obtained using a transgenic mouse line (Gene symbol: Crym) characterized by EGFP positive pyramidal neurons in cortical layers 5 and 6. Crym mice were obtained from the GENSAT Project at The Rockefeller University. No difference in results from these two groups was detected. The mice were decapitated following deep sodium pentobarbital (50 mg kg^−1^) anaesthesia, the brain was quickly removed, and 300 μm thick coronal cortical slices were cut in ice-cold solution using a custom made rotary slicer with circular blade (Specialty Blades Inc., Staunton, VA, USA). Slices were incubated at 37 ° C for ∼30 min and then maintained at room temperature (23–25 ° C). The standard extracellular solution used during recording contained (in mM): 125 NaCl, 25 NaHCO_3_, 20 glucose, 2.5 KCl, 1.25 NaH_2_PO_4_, 2 CaCl_2_ and 1 MgCl_2_, pH 7.4 when bubbled with a 5% CO_2_ gas mixture balanced with either 95% O_2_ or 95% N_2_ during short periods (∼5 min) of optical recordings. The transient hypoxic conditions dramatically reduced the sensitivity of neurons to photodynamic damage with no detectable effect on the physiological conditions of nerve cells supplied with phosphocreatine and ATP from the patch electrode^39^,^40^. Somatic whole-cell recordings in current clamp or voltage-clamp mode were made with 4–6 MΩ patch pipettes using a Multiclamp 700B amplifier (Axon Instruments Inc., Union City, CA, USA). Voltage-clamp recordings were made with series resistance compensation set at 70%. Synaptic currents recorded were corrected for inadequate space clamp using information from direct measurements of somatic voltage-clamp errors^23^. The pipette solution contained (in mM): 120 K-gluconate, 3 KCl, 7 NaCl, 4 Mg-ATP, 0.3 Na-GTP, 20 HEPES and 14 tris-phosphocreatin (pH 7.3, adjusted with KOH) and 0.8 mM of the voltage-sensitive dye JPW3028 (ref. 13). The pharmacological agents were obtained from Tocris (TTX, Nimodipine, DL-AP5). The somatic whole-cell recording data were not corrected for liquid junction potential. In experiments using wild-type mice, an attempt was made to identify layer 5 pyramidal cells with intact dendrites in one plane of focus close to the surface of the slice (to minimize light scattering) using infrared differential interference contrast (DIC) video microscopy. This approach is inefficient because thin dendrites cannot be well resolved under DIC. Thus, even after substantial experience, more than 50% of neurons loaded with the dye were subsequently found to be badly chosen (dendrites cut or not running parallel to the plane of focus). Selection of neurons with intact dendrites positioned close to the surface of the slice (<30 μm) was greatly facilitated using the Crym transgenic mouse line expressing EGFP in a subset of layer 5 pyramidal neurons. EGFP labelling allowed us to select appropriate neurons before dye loading by visually inspecting a slice under 488 nm excitation of EGFP fluorescence using spinning disk confocal microscopy mode at 5–20 frames per second (CSU-10 Yokogawa confocal scanner; Solamere Tech., Salt Lake City, UT). Dendrites of individual nerve cells were readily visible under fluorescence. EGFP fluorescence did not interfere with voltage‐sensitive dye signals due to the non‐overlapping emission spectra of these two fluorophores. In agreement with previous results on cerebellar Purkinje cells^41^, neurons with EGFP fluorescence had no detectable changes in electrical behaviour. The recordings were carried out from spines on superficial basal dendrites at different distances from the soma (range 30–120 μm). Labelling of pyramidal neurons with the voltage-sensitive dye was carried out by free diffusion from a somatic patch electrode in the whole-cell configuration. We used the most successful voltage probe for intracellular application, JPW3028, a close analogue of JPW1114 (ref. 42) with similar voltage sensitivity available from Invitrogen as D6923. The electrode tips were first filled with dye-free solution by applying negative pressure and then back filled with the solution containing the voltage probe (0.8 mM). The patch electrode was detached from the neuron by forming an outside-out patch after staining was accomplished, as determined by measuring resting fluorescence intensity from the soma. The optimal amount of staining was a compromise between high level of fluorescence and the damage that can be caused by prolonged dialysis of a neuron from the patch pipette. Following the staining period, the preparation was typically incubated for an additional 1.5–2 h at room temperature to allow the voltage-sensitive dye to spread into dendritic processes. Before optical recording, the cell was re-patched to obtain electrical recording using an electrode filled with dye-free intracellular solution

### Optical recording

The recording setup was built around a stationary upright microscope (AxioExaminer D1 with zoom tube (0.5–4 × ), Carl Zeiss Microscopy LLC or Olympus BX51; Olympus Inc., USA) equipped with three camera ports with a standard, high spatial resolution CCD camera for infrared DIC video microscopy (CCD-300-RC, Dage-MTI, Michigan City, IN, USA), a fast data acquisition camera with relatively low spatial resolution (80 × 80 pixels) and exceptionally low read noise (NeuroCCD-SM, RedShirtImaging LLC, Decatur, GA, USA), and a high spatial resolution CCD camera (1,392 × 1,024 pixels; Pixelfly-qe, PCO Imaging, Kelheim, Germany) mounted on a spinning disc confocal scanner (Yokogawa CSU-10). The spinning disc scanner was used to collect *z*-stacks of confocal images for detailed morphological reconstruction of dendritic spines. For two-photon glutamate uncaging, an additional dichroic mirror and IR blocking filter allowed introduction of 720 nm light (see below). Optical recording was carried out in the wide-field epi-fluorescence microscopy mode because the superior spatial resolution of confocal and two-photon fluorescence microscopy techniques are difficult to utilize in Vm-imaging. Due to high fractional shot noise related to the small number of emitted photons^43^,^44^,^45^, these methods have a poor *S*/*N*. The fluorescent image of the stained neuron was projected by a water immersion objective onto the fast data acquisition CCD positioned in the primary image plane. We used either a × 63/1.0 numerical aperture (NA), Carl Zeiss objective with a × 1.6 zoom tube or a × 100/1.0 NA Olympus objective without zoom tube. A laser was used as a source for excitation light in place of a conventional Xenon arc lamp to increase the sensitivity of Vm-imaging by providing a monochromatic excitation light at the red wing of the absorption spectrum to maximize Vm sensitivity of the dye^18^,^43^,^46^. In addition, the laser allowed us to increase the intensity of the excitation light beyond the level that can be achieved by an arc lamp. We used a 500 mW diode-pumped, continuous wave laser emitting at 532 nm (MLL532, Changchun New Industries Optoelectronics Tech. Co., Ltd., Changchun, China). The laser beam was coupled to the microscope via a light guide and a single-port epifluorescence condenser (TILL Photonics GmbH, Gräfelfing, Germany). Excitation light was reflected to the preparation by a dichroic mirror (560 nm) and the fluorescence light was filtered by a band pass emission filter (FF01-720; 720 nm blocking edge BrightLine, Semrock). A CCD frame (80 × 80 pixels) corresponded to a field of 18 × 18 μm in the object plane.

### Two-photon uncaging of glutamate

The voltage imaging setup was integrated with an ultra-fast pulsed titanium-sapphire laser tuned to 720 nm for glutamate uncaging (Chameleon Ultra, Coherent Inc.). The expanded beam of the laser was directed to the scan mirror and passed to the back opening of the objective through a dichroic mirror (FF705, Semrock) which reflected the voltage-sensitive dye fluorescence to a CCD camera. The light intensity of the laser was controlled by a Pockells cell (Model 350-80, Conoptics Inc.). The diffraction limited 720 nm stationary light spot was positioned in the centre of the field of view. We found that commonly used MNI caged glutamate was possible to use in combination with voltage-imaging if applied in high concentration (∼20 mM) from an extracellular glass pipette. However, the 720 nm light intensities required for adequate release of glutamate often resulted in significant bleaching of the membrane bound voltage-sensitive dye in the spine head contaminating the eEPSP signal. Bath applied DNI-glutamate TFA (5 mM) provided by Femtonics KFT (Budapest, Hungary) has ∼7 times higher two-photon uncaging efficiency^11^ and was more favourable to use. There were no differences in the results obtained with the two compounds both in terms of spatial selectivity and the obtained current responses. The spine head was positioned close (∼0.5 μm) to the stationary uncaging site in the centre of the field of view using a motorised movable top plate (*x*–*y* resolution 0.02 μm, Scientifica, UK) and an uncaging light pulse (∼10 mW, 0.45–0.5 ms) was applied. By fine tuning the intensity of the uncaging light pulse, it was possible to closely approximate the amplitude and the time course of miniature EPSCs.

### Data analysis

Subthreshold eEPSP signals were recorded typically for 40 ms at a frame rate of 2 kHz. AP signals were recorded for 10 ms at 5 kHz at near physiological temperature of 32–34 C° or at 2 kHz at room temperature kept at 25–27 °C. Analysis and display of data were carried out using the NeuroPlex programme (RedShirtImaging) written in IDL (Exelis Visual Information Solutions, Boulder, CO) and custom Visual Basic routines. Under low light conditions, background fluorescence becomes a significant determinant of Δ*F*/*F* signal size. Raw data were first corrected for this effect by subtracting the average background fluorescence intensity determined from an unstained area on the slice. Subsequently, signal alignment software was used to correct for temporal jitter in AP initiation as well as for possible small movements of the preparation during averaging^47^. In the temporal domain, AP signals were aligned by cross-correlation of the electrically recorded APs in each trial to the reference signal acquired at the start of averaging. In the spatial domain, camera images were aligned in two dimensions offline by image cross-correlation to compensate for possible small lateral movements of the preparation^47^. Correct focus of the image in the *z*-dimension was verified before each individual trial; small adjustments were often necessary. The spatially and temporally aligned signals were averaged and slow changes in light intensity due to bleaching of the dye were corrected by dividing the data by an appropriate dual exponential function derived from the recording trials with no stimulation^48^. Dual exponential fitting requires an algorithm for least-squares estimation of nonlinear parameters. We used the Levenberg–Marquardt algorithm. The waveform of the AP signal was reconstructed from a set of data points using Cubic Spline Interpolation, a piecewise continuous curve passing through each data point^47^. Subthreshold optical signals were calibrated on an absolute scale (in mV) by normalizing to an optical signal from a bAP which has a known declining amplitude along basal dendrites, as previously determined by patch-pipette recordings^12^,^17^. We experimentally confirmed (Supplementary Fig. 1) the previously reported result^12^,^18^ showing that this methods of calibration produce the same results as normalizing signals to optical recordings corresponding to long hyperpolarizing pulses delivered to the soma which attenuate relatively little as they propagate along dendrites^12^,^17^,^19^.

### Computational modelling

The spine model, constructed with NEURON simulator^49^, represented the spine as a cylinder (head) attached to the distal end of another cylinder (neck) (equivalent electrical circuit in Supplementary Fig. 3). Anatomical properties of spines (neck diameter 0.18 μm; neck length 1 μm) were adjusted to match the data from recent supra-resolution microscopy measurements from living spines in brain slices^7^,^31^,^50^. Resting potential was −70 mV unless otherwise noted, and numerical integration used NEURON’s default implicit Euler method, with d*t* 0.025 ms. In all simulations the spine head and neck were treated as a single compartment which was quite sufficient for spatial accuracy given the range of anatomical and biophysical properties that was explored. The model parameters are based on ref. 12 with cytoplasmic resistivity (*R*_a_) 100 Ω cm, specific membrane capacitance (*C*_m_) 1 μf cm^−2^ and membrane leak conductance (g_pas) 1/20,000 S cm^−2^. To generate synaptic current, we attached a biexponential conductance change synapse model to the spine head with parameters *τ*_1_ 0.2 ms, *τ*_2_ 3 ms, peak conductance 0.291 nS, reversal potential 0 mV to approximate the experimentally determined current amplitude and time course corresponding to a unitary EPSP amplitude in the soma in the range 0.2–0.8 mV (refs 21, 51, 52). Dendritic diameters of the reconstructed layer 5 pyramidal neuron from the somatosensory cortex were determined using confocal microscopy of live nerve cells labelled with the dye used for voltage imaging (JPW3028). As this lipophilic dye partitions into the membrane lipid bilayer, it was possible to accurately determine dendritic diameters down to the diffraction limit of resolution (∼0.3 μm) by recording the profile of light intensity across dendritic branches (Supplementary Fig. 4). Accurate measurements of dendritic diameters are critical because electrical role of spines is a function of the ratio of spine neck resistance (*R*_neck_) and dendritic input impedance (*Z*_dendrite_). Because *Z*_dendrite_ is a steep function of diameter, errors in *Z*_dendrite_ estimates can lead to misinterpretation of both experimental and modelling results. Morphologies of mouse neocortical pyramidal cells were obtained from NeuroMorpho.org, specifically cell 070502-exp2-zB from the Krieger archive. The models used in these simulations omitted the axon. Source code for these models will be made available from ModelDB https://senselab.med.yale.edu/modeldb/.

## Additional information

**How to cite this article:** Popovic, M. A. *et al*. Electrical behaviour of dendritic spines as revealed by voltage imaging. *Nat. Commun.* 6:8436 doi: 10.1038/ncomms9436 (2015).

## Supplementary Material

Supplementary Information Supplementary Figures 1-4, Supplementary References
