# Supplementary material for: Electrical behaviour of dendritic spines as revealed by voltage imaging
Source: Nat Commun. 2015 Oct 5;6:8436. doi: 10.1038/ncomms9436 (PMC4594633; doi:10.1038/ncomms9436)
Supplement: Supplementary Information — Supplementary Figures 1-4, Supplementary References [file ncomms9436-s1.pdf]

Popovic et al. Electrical behavior of dendritic spines

Supplementary information

Supplementary figure 1

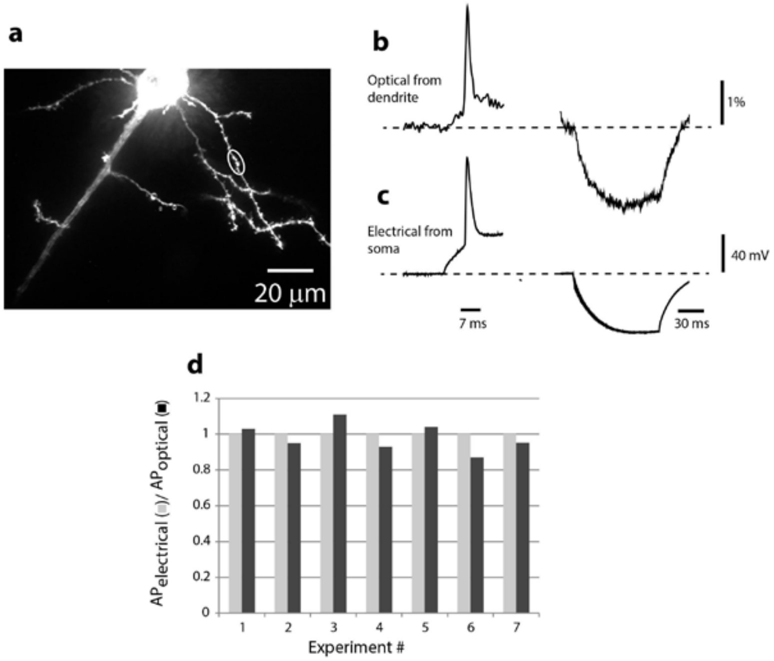

Calibration of optical signals in terms of membrane potential. a, Fluorescence image of a layer 5 pyramidal neuron. **b,** Optical bAP signals (left) and long hyperpolarizing pulse (right) from a 10 μm long section of a basal dendrite (white ellipse in (a)). **c,** lectrode recordings of same signals from soma. The amplitude of the optically recorded bAP normalized to the 60 mV steady state optical signal, is 104 mV while the expected value of the bAP at a distance 30 μm from the soma is in the range of 85 - 105 mV[1,2]. **d,** Optically recorded bAP amplitudes (n=7) calibrated using signals corresponding to hyperpolarizing pulses (black bars) normalized to the average bAP amplitude of 84 mV derived from published measurements with dendritic patch electrodes within the same range of distances (10 to 50 μm) from the soma (gray bars)[1]. The variability in optically recorded bAP amplitudes (±10 mV) is similar to the variability in electrode recordings (±10 mV)[1].

Supplementary figure 2

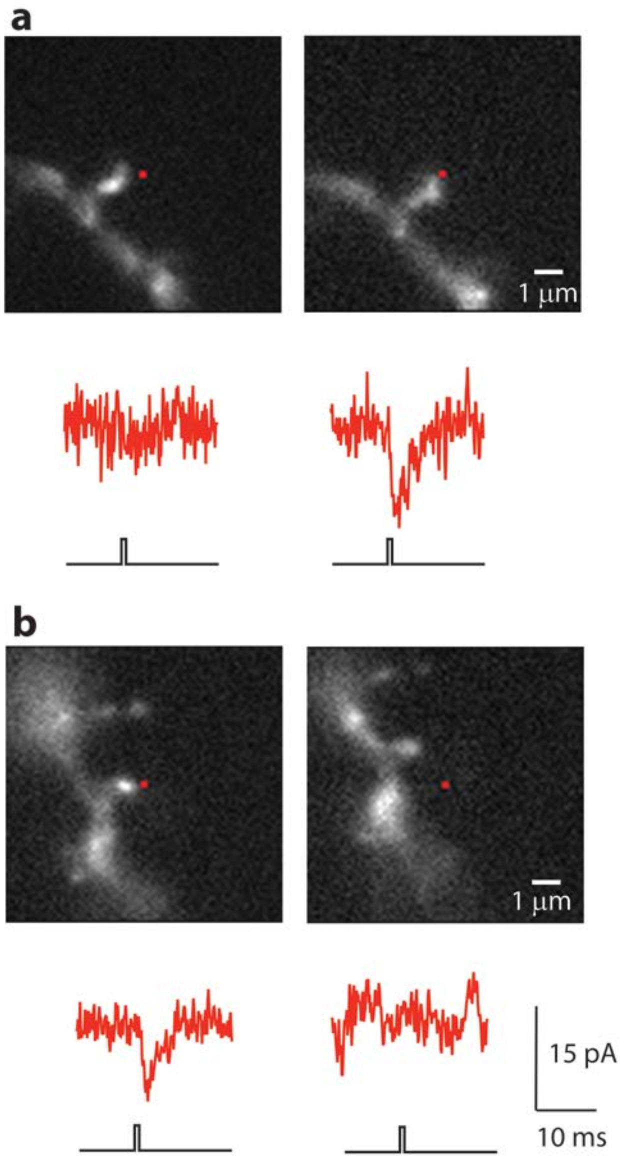

Spatial selectivity of 2-photon glutamate uncaging. a, Single frame fluorescence image of a spine in recording position. Red dot: uncaging site. Synaptic current response to focal application of glutamate (lower red traces) is sensitive to the position of the uncaging site relative to spine head at the sub-micrometre scale. Black traces indicate uncaging time. **b**, Synaptic current response to uncaging glutamate at the spine head (left). Uncaging at location without spine (right) at the same distance from the dendrite elicits no response.

Supplementary figure 3

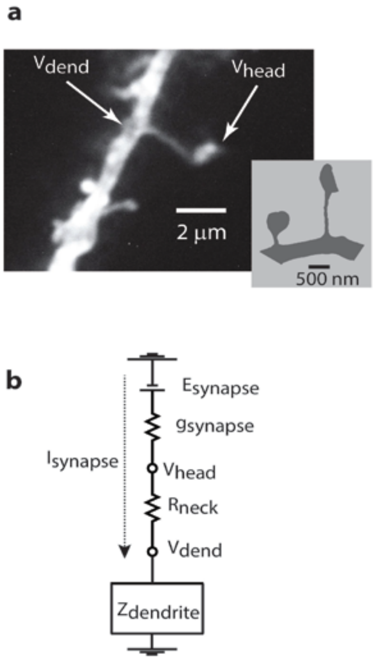

c

$$V_{spine} = I_{syn}(R_{neck} + Z_{dend})$$

$$V_{dend} = I_{syn}Z_{dend}$$

$$V_{spine} = I_{syn}R_{neck} + I_{syn}Z_{dend}$$

$$I_{syn}R_{neck} = V_{spine} - V_{dend}$$

$$R_{neck}\int_0^t I_{syn}dt = \int_0^t [V_{spine}(t) - V_{dend}(t)]dt$$

$$\int_0^t I_{syn}dt = Q_{syn}$$

$$Q_{syn} = \frac{Q_{clamp}}{K_{s-d}}$$

$$K_{s-d} = \frac{Q_{clamp}}{Q_{syn}}$$

$$R_{neck} = \frac{\int_0^t [V_{spine}(t) - V_{dend}(t)]dt}{\frac{Q_{clamp}}{K_{s-d}}}$$

$$Z_{dend} = \frac{\int_0^t V_{dend}dt}{\frac{Q_{clamp}}{K_{s-d}}}$$

Computation of Rneck and Zdendrite. a, Fluorescence image of a spine attached to a dendrite obtained from a z-stack of confocal images. Inset: More precise spine morphology and dimensions, obtained by supra-resolution fluorescence STED microscopy; the outline corresponds to actual living spines as reported by Tonnesen et al. (2014). **b,** Equivalent electrical circuit of a spine attached to a dendrite. **c,** Derivation of equations for calculating R_{neck} and $Z_{dendrite}$ using Ohm's law and Kirchhoff's law applied to the equivalent electrical circuit in B. $V_{dendrite}$ - local dendritic membrane potential; V_{spine} - membrane potential of the spine head; $I_{synapse}$ – synaptic current; R_{neck} – electrical resistance of spine neck; $Z_{dendrite}$ – impedance of parent dendrite; Q_{clamp} – total recorded charge transfer; Q_{syn} – estimated total charge transfer at the synaptic site; K_{s-d} – correction factor based on experimentally determined somatic voltage-clamp errors[3].

Supplementary figure 4

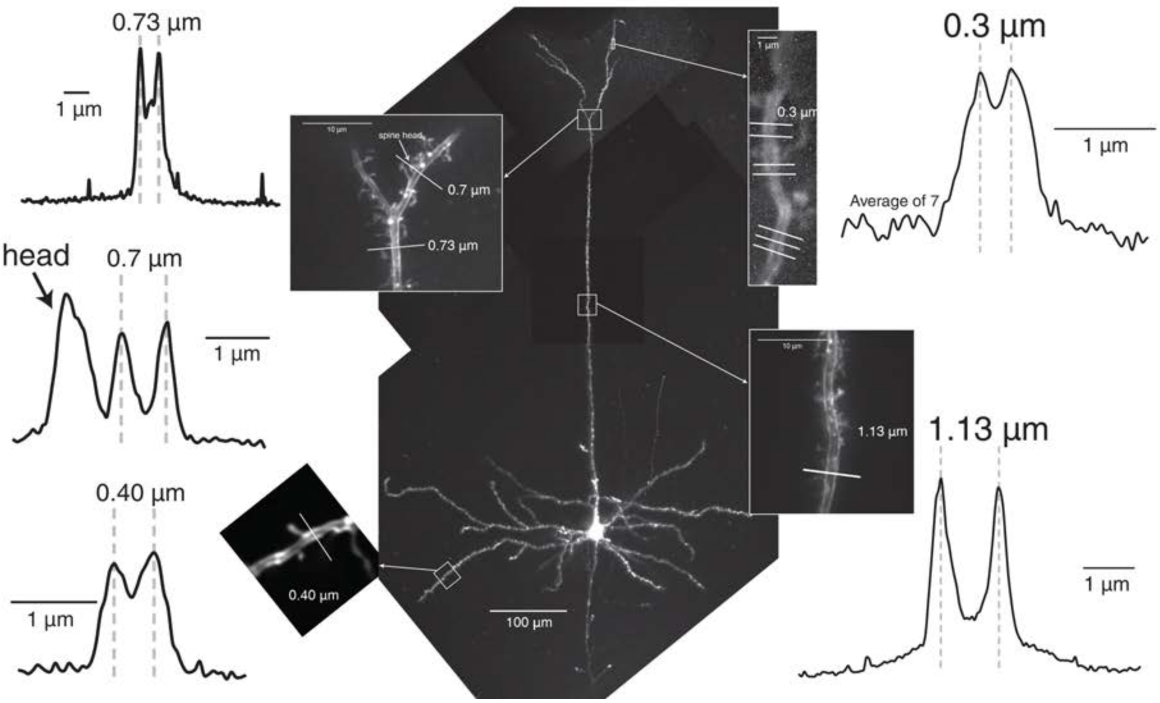

Dendritic diameters. Confocal image of a layer 5 pyramidal neuron from mouse somatosensory cortex. Measurement of diameters of different dendritic compartments. Average diameters were: basal dendrites, 0.4 µm; primary dendrites, 1 to 2 µm; distal apical tuft branches, 0.3 µm. In the model, the correct dendritic diameters are critical determinants of the passive electrical behaviour of neurons.

1. Nevian, T., Larkum, M. E., Polsky, A. & Schiller, J. Properties of basal dendrites of layer 5 pyramidal neurons: a direct patch-clamp recording study. *Nat. Neurosci.* **10,** 206–14 (2007).

2. Palmer, L. M. & Stuart, G. J. Membrane potential changes in dendritic spines during action potentials and synaptic input. *J. Neurosci.* **29,** 6897–903 (2009).

3. Williams, S. R. & Mitchell, S. J. Direct measurement of somatic voltage clamp errors in central neurons. *Nat. Neurosci.* **11,** 790–8 (2008).
